# The impact of echinococcosis interventions on economic outcomes in Qinghai Province of China: Evidence from county-level panel data

**DOI:** 10.3389/fvets.2023.1068259

**Published:** 2023-03-17

**Authors:** Jinshan Cai, Kefan Yang, Qihui Chen, Quanbang Zhao, Jing Li, Sen Wang, Lin Yang, Yumei Liu

**Affiliations:** ^1^Veterinary Public Health Department, Qinghai Center for Animal Disease Prevention and Control, Xining, Qinghai, China; ^2^The Research Key Laboratory for Echinococcosis of Qinghai Province, Xining, Qinghai, China; ^3^College of Economics and Management, China Agricultural University, Beijing, China; ^4^China Animal Disease Control Center, Beijing, China

**Keywords:** echinococcosis, interventions, economic evaluation, dynamic difference-in-differences model, China

## Abstract

**Introduction:**

Echinococcosis can incur substantial economic losses for the livestock industry by causing organ condemnation, delayed growth, and reduced meat and wool output and quality in sheep and cattle, as well as increased surgery costs, hospital care, and decreased productivity in humans. Yet echinococcosis could be prevented and controlled by interventions, such as dog management and deworming, lamb vaccination, slaughter management, and training and public education.

**Methods:**

Exploiting temporal and spatial variations in the number of intervention measures implemented in 39 counties in Qinghai province of China in 2015–2020, this study assesses the economic impact of echinococcosis interventions using a dynamic difference-in-differences model.

**Results:**

The results suggest that echinococcosis interventions brought about substantial economic gains measured by per capita net income of rural residents and per capita gross output of animal husbandry. These economic gains are greater in non-pastoral counties (with a gain in per capita net income of rural residents of 3,308 yuan and a gain per capita gross output of animal husbandry of 1,035 yuan) than in pastoral counties (with a gain in per capita net income of rural residents of 1,372 yuan and a gain per capita gross output of animal husbandry of 913 yuan). They are also greater in counties with echinococcosis infection level-2 (with a human infection rate of 0.1–1% or a dog infection rate of 1–5%) than infection level-1 counties (with a human prevalence rate ≥1% or a dog infection rate ≥5%).

**Discussion:**

Not only will these economic gains encourage livestock farmers to strengthen their echinococcosis prevention and control practices, but they will also inform public policy on zoonotic disease prevention and control in China and other countries alike.

## Introduction

Echinococcosis (also known as “hydatidosis” or “hydatid disease”) is a major pathogenic and lethal parasitic zoonosis caused by the larvae of Echinococcus granulosus parasitizing in humans or cattle and sheep (1). As with many other zoonotic diseases, echinococcosis can cause serious harm to livestock and human health (2–4), thereby incurring substantial economic losses (5–7). In livestock, echinococcosis can lead to reduced birth rate, delayed growth, lowered yield and quality of meat and wool, destructed viscera, and even death, undermining the production efficiency of the livestock industry (3, 8, 9). In humans, economic losses may result from surgical treatments, hospital care, and forgone income due to impaired labor productivity (3).

China is one of the countries that have a high prevalence of echinococcosis, particularly in the West, North, and Northwest parts, home to eight provincial-level administrative units with vast agricultural and pastoral lands (Qinghai, Sichuan, Gansu, Yunnan, Xinjiang, Ningxia, Inner Mongolia, and Tibet) (10).

As one of China's five grassland animal husbandry bases, Qinghai is one of the provinces that are most seriously impacted by echinococcosis, with cystic echinococcosis caused by larvae of Echinococcus granulosus being the primary disease. In Qinghai, dogs serve as the primary definitive host for Echinococcus granulosus, harboring viscera from infected cattle and sheep, whose small intestines are homes to mature parasitizing tapeworms (11–13). Cattle and sheep serve as intermediate hosts by ingesting eggs shed by the definitive host (3, 5, 13).

A series of inspections conducted by the Qinghai Center for Animal Disease Prevention and Control (QACDC) in five counties (Zeku, Chengduo, Delingha, Gande, and Guinan) in 2,000 tested a total of 550 cattle, 4,631 sheep, and 306 dogs, revealing echinococcosis granulosus infection rates of 60.36, 53.14, and 35.95%, respectively.

An epidemiological survey of echinococcosis (detected by the ultrasound method) conducted in 2012, by the Qinghai Center for Disease Prevention and Control (QCDC), discovered that the average human infection rate in the province was 0.63% and as high as 12.38% in some counties.^1^

To effectively prevent and control echinococcosis, a project entitled “*Action Plan for Prevention and Control of Echinococcosis in Qinghai Province (2016–2020)*” (—referred to as the “*Action Plan (2016–2020)*” below) financed by China's central government was launched in Qinghai at the end of 2015. The Qinghai Department of Agriculture and Rural Affairs implemented four intervention measures in Qinghai's counties, with a focus placed on controlling the source of infection:

*Measure 1: Dog management and deworming*. This measure includes dog registration, deworming, feces disposal after deworming, and controlling the number of dogs. Among these activities, dog registration involves creating dog deworming registration cards, recording all domestic (shepherd) dogs in the project area, putting an identity card around each dog's neck, and registering stray dogs within a monitoring unit (usually a village); deworming is to feed each dog with one or two praziquantel tablets (specification: 0.2 g/tablet) once a month, which ought to be recorded on the dog's deworming registration card; feces disposal is to collect dogs' feces and bury them deep under the ground or incinerate them within 5 days after deworming, which prevents Echinococcus eggs from contaminating the environment; finally, measures taken to control the number of dogs include culling stray dogs, as well as sterilizing and breeding them in captivity.

*Measure 2: Lamb vaccination*. This measure uses the Recombinant Hydatids Subunit Vaccine produced by Chongqing AULEON Biologicals CO., LTD in China to immunize lambs, especially in pastoral areas where the prevalence of echinococcosis is high.

*Measure 3: Slaughter management*. This measure includes strengthening and supervising slaughters' performance in applying hazard-free treatments (e.g., high temperature, high pressure, incineration, or deep burial) to deal with diseased organs and advising farmers to avoid feeding their dogs with diseased organs.

*Measure 4: Training and public education*. This involves popularizing the knowledge about echinococcosis prevention and control to relevant local entities, including schools, ranches, villages, herders, monasteries, enterprises, and local governments. Bilingual materials (in both Chinese and Tibetan) are distributed through public media, such as brochures, television news, special interviews, promotional videos, social media accounts, and other channels. Meanwhile, training on echinococcosis prevention and control practices is provided to herders and local epidemic prevention personnel.

By 2020, achievements in echinococcosis prevention and control in Qinghai had been remarkable. The number of annual newly diagnosed cases of echinococcosis in humans declined from 0.63 per 100 people in 2012 to 0.17 per 100 people in 2021.^2^ Echinococcosis infection rates among animals also decreased dramatically: those in dogs, cattle, and sheep decreased from 48.12, 15.24, and 19.59% in 2015, respectively, to 2.28, 1.59, and 1.57% in 2020. These achievements, in fact, echo the successful experiences of a number of countries. For example, the deworming treatment of domestic dogs adopted in Argentina, Chile, and Uruguay reduced the echinococcosis infection rate from a striking 71% in 1979 to a mere 0.35% in 1997 (14). A large number of studies have been conducted to investigate the feasibility of these measures, their effectiveness in reducing infection rates or eliminating echinococcosis, and risk factors for infection among intermediate and definitive animal hosts (13, 15, 16). However, few have evaluated the *economic* impact of echinococcosis prevention and control interventions, especially in the context of China.

The recently implemented “*Action Plan (2016–2020)*” in Qinghai provides a unique opportunity to fill this gap.

Motivated by the significant drops in infection rates, the present study intends to assess the *economic* effect of echinococcosis prevention and control interventions in Qinghai's counties, measured by the increases and growth rates of rural residents' per capita net income and per capita gross output of animal husbandry at the county level. In particular, we compare changes in economic indicators before and after echinococcosis interventions between counties that implemented all four intervention measures and those that implemented only three—i.e., a difference-in-differences analysis that addresses the influence of potential time-invariant confounding factors. The findings of this study will not only help encourage farmers to strengthen their echinococcosis prevention and control practices but also inform public policy on zoonotic disease prevention and control.

## Materials and methods

### Study area and data

The study area of this paper, Qinghai, is a landlocked province in Northwest China. It is the fourth-largest province of China by area and the third-smallest by population. Located mostly on the Tibetan Plateau, its average elevation is more than 3,000 m above sea level. Mainly constrained by its remote location, unfavorable geographical conditions, and lack of transportation infrastructure, Qinghai's economy is amongst the least developed in China. Its nominal GDP in 2021 was only 334.7 billion yuan,^3^ the second-lowest among China's provincial-level units, amounting to about 0.29% of China's national GDP.^4^ Outside of the provincial capital, Xining City, Qinghai's economy is primarily agrarian, divided mainly by the Riyue Mountain into pastoral zones in the west and agricultural zones in the east.

The data analyzed in this study were drawn from the *Statistical Manuals of Agriculture and Animal Husbandry of Qinghai Province (2014–2020)* and the echinococcosis monitoring database (on all livestock farms in each county) from 2016 to 2020 compiled by each county's Animal Husbandry and Veterinary Bureau in Qinghai. The study sample includes 39 out of 42 counties in Qinghai, of which nine are categorized as agricultural zones, 24 as pastoral zones, and six as agro-pastoral zones (Table 1).^5^

**Table 1:** Characteristics of sampled counties in 2015.

**Infection level**	**Number of counties**	**Average altitude (m)**	**Type of areas (by dominant agricultural activity)**			**Interventions (in 2016)**	
			**Number characterized as agricultural zones**	**Number characterized as agro-pastoral zones**	**Number characterized as pastoral zones**	**Number with three measures**	**Number with four measures**
1	32	3,244	5	4	23	14	18
2	7	2,500	4	2	1	6	1
Total	39		9	6	24	20	19

Source: Calculated based on data from the Statistical Manuals of Agriculture and Animal Husbandry of Qinghai Province (2014–2020), complied by the Qinghai Agriculture and Animal Husbandry Department.

The choice of the study sample was mainly based on data availability. For echinococcosis prevention and control purposes, Qinghai's 42 counties were categorized into four levels of echinococcosis infection: 32 counties were categorized as echinococcosis infection level-1 (with a human infection rate ≥1% or a dog infection rate ≥5%), seven as level-2 (with a human infection rate of 0.1–1% or a dog infection rate of 1–5%), and three as level-4 (with a human infection rate < 0.05% or a dog infection rate < 0.1%). But because no monitoring data were available for the three level-4 counties due to their remoteness and poor infrastructure conditions, our sample includes the 39 level-1 and level-2 counties (Table 1).

Of the 39 sampled counties, 19 are located in pastoral areas with a relatively high altitude (mean = 3,632 m), where the economy is less developed and echinococcosis infection is relatively more severe. In the starting year of the *Action Plan (2016–2020)*, 2016, these 19 counties implemented all four echinococcosis prevention and control measures described in the Introduction section. The other 20 counties are located at a lower altitude (mean = 2,615 m), where the economy is relatively more developed, and echinococcosis infection is less severe. These 20 counties implemented only three echinococcosis prevention and control measures in 2016 (i.e., dog management and deworming, slaughter management, and training and public education, *but not lamb vaccination*) due to limited central financial resources. As the *Action Plan (2016–2020)* unfolded, some of these 20 three-measure counties later implemented lamb vaccination sometime before 2020.

### Economic impact assessment methods

#### Comparative analysis

To assess the economic impact of echinococcosis interventions in Qinghai, we performed two sets of statistical analyses. The first set of analyses computes the *increases* and *growth rates* of two economic indicators of primary interest (i.e., per capita net income of rural residents and per capita gross output of animal husbandry) to capture the economic gains of echinococcosis interventions from 2015 to 2020 for each county, and then compares them across subgroups of counties. Following Liu et al. (7), we calculated the increased value (*Y*) and the growth rate (*g*) of a given economic indicator (*Y*) as follows:


(1) $$\begin{array}{l} Increased\;value\;\left(Y\right)=Y_{2020}-Y_{2015}, \end{array}$$

(2) $$\begin{array}{l} Growth\;rate\;\left(g\right)=\frac{Y}{Y_{2015}}\times 100\%. \end{array}$$

All economic measures involved have been deflated by the county-level rural consumer price index (CPI), using 2014 as the base year. The set of comparative analyses is performed for Qinghai as a whole and then separately for county subgroups defined by the number of intervention measures (i.e., those with all four measures implemented vs. those with only three).

#### Dynamic difference-in-difference analysis

The second set of statistical analyses involves more advanced regression analyses. The temporal differences in the implementation of lamb vaccination across counties provide a unique opportunity to rigorously evaluate the economic impact of echinococcosis interventions (lamb vaccination, in particular) based on a dynamic difference-in-differences (DID) model, which has the advantage of being able to eliminate time-invariant confounding factors (17–19). In the DID analysis, counties that implemented all four measures in a given year during the project period (2016–2020) are used as the treatment group; those with only three measures implemented (without lamb vaccination) in that year are used as the comparison group.

Exploiting temporal and spatial variations in the number of intervention measures implemented in different counties (i.e., four vs. three, with or without lamb vaccination), we estimate the following dynamic difference-in-differences (DID) model to assess the economic impact of echinococcosis interventions (lamb vaccination, in particular):


(3) $$\begin{array}{l} Y_{it}=\alpha +\beta D_{it}+\gamma X_{it}+C_{i}+T_{t}+\varepsilon_{it},\;i=1,\cdots \;,\;39;\\ t=2015,\cdots \;,2020; \end{array}$$

In this model, the outcome variable, *Y*_*it*_, is an economic indicator of interest in county *i* in year *t*. The key explanatory variable, *D*_*it*_, is a dummy variable that equals one if the *i*th county implemented all four echinococcosis intervention measures in year *t* and equals zero otherwise. *C*_*i*_ and *T*_*t*_ are, respectively, county and year fixed effects, which control for county-level time-invariant confounding factors and the general trend of economic development. *X*_*it*_ is a set of time-varying county-level variables that serve as additional controls, including the proportions of illiterate labor, labor with high school education or above, and off-farm labor, as well as the numbers of villages with roads, telephones, and running water. Finally, ε_*it*_ is the disturbance term.

If Equation (3) is adequately specified, the coefficient β captures the impact of echinococcosis interventions (lamb vaccination, in particular) on *Y*. More intuitively, β measures whether the outcome variable *Y* grew faster during the intervention period (2016–2020) relative to their pre-intervention levels in counties that implemented all four intervention measures (the “treatment” group), compared with those counties implementing only three (the “comparison” group). A positive and statistically significant estimate of β will provide strong evidence that echinococcosis interventions implemented by the *Action Plan (2016–2020)* (in particular, lamb vaccination) indeed exert a positive effect on the economic indicators of interest (17).

#### Parallel trend test

The key assumption for the DID model (Equation 3) to yield unbiased estimates of β is the so-called “parallel-trend” assumption (20, 21). That is, the time trends of outcome measures in the “treatment” group (counties with all four measures implemented) and the “comparison” group (counties with only three measures implemented) should be similar (“parallel”) *in the absence* of echinococcosis interventions (lamb vaccination). With data from two pre-intervention periods available, we can assess the validity of this assumption by formally testing whether the outcome measures in two groups of counties indeed had parallel trends before the intervention. More specifically, we follow Chen et al. (22) and estimate the following model using data from the two pre-intervention years (2014 and 2015):


(4) $$\begin{array}{l} Y_{is}=\alpha +\delta \left(D_{is}^{0}\times d_{2015}\right)+\gamma X_{is}+C_{i}+T_{s}+\varepsilon_{is}, \end{array}$$

where $D_{is}^{0}$ is a dummy variable equaling one if the *i*th county in the *s*th year *would implement all four measures from 2016* to 2020 and zero if only three would be implemented (without lamb vaccination); *d*_2015_ is a dummy variable for the year 2015 (the placebo “post-intervention” year). If the parallel-trend assumption is plausible, one would expect the coefficient of the interaction term, δ, to be small and statistically insignificant, i.e., there were no significant treatment-comparison differences in the time trends before the intervention.

All statistical analyses discussed in this section will be performed in STATA SE 14.

## Results

### Descriptive analysis

Table 2 provides summary statistics of the two economic indicators of primary interest, i.e., per capita net income of rural residents and per capita gross output of animal husbandry in 2016, the starting year of the *Action Plan (2016–2020)* for the sampled counties. The average per capita net income of rural residents among all 39 sampled counties was 8,359.6 yuan. It was higher in counties with three intervention measures (9,317.7 yuan), counties at infection level-2 (9,563.8 yuan), and counties in agro-pastoral zones (9,918.7 yuan). The average per capita gross output of animal husbandry among sampled counties was 6,435.2 yuan. In contrast to the pattern observed for the first indicator (per capita rural net income), this indicator was higher in counties with all four intervention measures (7,930.1 yuan), counties with infection level-1 (6,907.1 yuan), and counties in pastoral zones (8,617.1 yuan). The main aim of this study is to assess how the implementation of the *Action Plan (2016–2020)* led to changes in the above two economic indicators across counties with different characteristics.

**Table 2:** Economic characteristics of sampled counties in 2016 (the starting year of echinococcosis interventions).

**Indicators**			**Mean**	**SD**	**Min**	**Max**
Per capita net income of rural residents (yuan)	All counties		8,359.6	2,298.3	4,401.8	14,842.3
	Interventions	With 3 measures	9,317.7	1,978.9	5,462.8	14,842.3
		With 4 measures	7,351.0	2,219.0	4,401.8	12,354.0
	Echinococcosis infection level	Level-1	8,096.2	2,126.4	4,401.8	12,354.0
		Level-2	9,563.8	2,834.0	6,481.1	14,842.3
	Type of area	Agricultural	8,670.6	312.4	7,943.1	9,068.6
		Agro-pastoral	9,918.7	2,871.1	7,549.5	14,842.3
		Pastoral	7,853.2	2,441.6	4,401.8	12,354.0
Per capita gross output of animal husbandry (yuan)	All counties		6,435.2	5,098.9	1,426.9	22,033.9
	Interventions	With 3 measures	5,015.0	4,944.2	1,426.9	21,837.5
		With 4 measures	7,930.1	4,948.0	2,665.5	22,033.9
	Echinococcosis infection level	Level-1	6,907.1	5,343.7	1,426.9	22,033.9
		Level-2	4,278.2	3,241.1	1,940.3	11,250.6
	Type of area	Agricultural	2,635.4	1,012.9	1,426.9	3,942.6
		Agro-pastoral	3,407.4	1,043.6	2,412.1	5,208.6
		Pastoral	8,617.1	5,417.7	2,665.5	22,033.9

Source: Calculated based on data from the Statistical Manuals of Agriculture and Animal Husbandry of Qinghai Province (2014–2020), complied by the Qinghai Agriculture and Animal Husbandry Department.

#### Changes in economic indicators before and after echinococcosis interventions

To assess the economic impact of echinococcosis interventions implemented by the *Action Plan (2016–2020)*, we first calculated the increases in rural residents' per capita net income and per capita gross output of animal husbandry from 2015 to 2020 (Equation 1), as well as their growth rates (Equation 2), for the 39 sampled counties. Taking Qinghai as a whole, per capita net income of its rural residents increased from 7,778.7 yuan in 2015 to 11,115.0 yuan in 2020, with an overall growth rate of 42.9%. During the same period, the gross output of animal husbandry per capita increased from 6,369.1 to 8,235.5 yuan, with a growth rate of 29.3%. These substantial increases suggest significant economic gains generated by echinococcosis interventions.

#### Comparison between counties with all four intervention measures and those with three

If the *Action Plan (2016–2020)* was indeed effective, one would expect the number of intervention measures implemented to play a role in generating economic gains from 2015 to 2020. Table 3 reports the increases in the two primary economic indicators and their respective growth rates for counties with all four measures implemented (column 2) and those with only three implemented (column 3). The table shows that from 2015 to 2020, rural residents' per capita net income and its growth rate were, respectively, 187.4 yuan and 20.7 percentage points *higher* in four-measure counties than in three-measure counties. The difference in growth rates between the two groups of counties is statistically significant at the 8% level (column 4), partly reflecting the positive effect of more intervention measures. Turning to the second economic indicator of interest, Table 3 indicates that the increases in per capita gross output of animal husbandry and its growth rate were somewhat lower in four-measure counties than in three-measure counties. However, the difference in neither dimension is statistically significant (column 4).

**Table 3:** Changes and growth rates of economic indicators for four- and three-measure counties from 2015 to 2020.

**Indicators**	**(1) Summary statistics**	**(2) Counties with four measures implemented**	**(3) Counties with three measures implemented**	**(4) *t*-tests for differences between (2) and (3) (*P*-value)**
Increase in per capita net income of rural residents (yuan/year)	Mean	3,432.4	3,245.0	0.7854
	SD	2,724.5	1,352.8	
Growth rate of per capita net income of rural residents (%)	Mean	59.5	38.8	0.0800
	SD	48.5	16.6	
Increase in per capita gross output of animal husbandry (yuan/year)	Mean	1,752.0	1,975.1	0.7226
	SD	1,129.9	2,484.8	
Growth rate of per capita gross output of animal husbandry (%)	Mean	22.8	50.3	0.2571
	SD	11.0	102.3	
*N*		19	20	

Source: author's calculation.

Further tracing out the yearly evolution of rural residents' per capita net income from 2016 to 2020, Figure 1 depicts clear upward trends in both per capita net income (left) and its growth rate (right) for both four-measure counties and three-measure counties.^6^ Importantly, note that while three-measure counties (upper curves) outperformed their four-measure counterparts (bottom curves) in both dimensions at the beginning of the intervention period of the *Action Plan* (2016), the latter group caught up and eventually surpassed the former by the end of the evaluation period (2020). More specifically, the difference in rural residents' per capita net income between four-measure and three-measure counties increased from −1,967 yuan in 2016 to 382 yuan in 2020. Correspondingly, the difference in the growth rate of this indicator increased from −25.3 percentage points in 2016 to 4.9 percentage points in 2020. These comparisons highlight a stronger momentum gained by counties with all four intervention measures implemented during the evaluation period over those with only three measures implemented.

**Figure 1:**
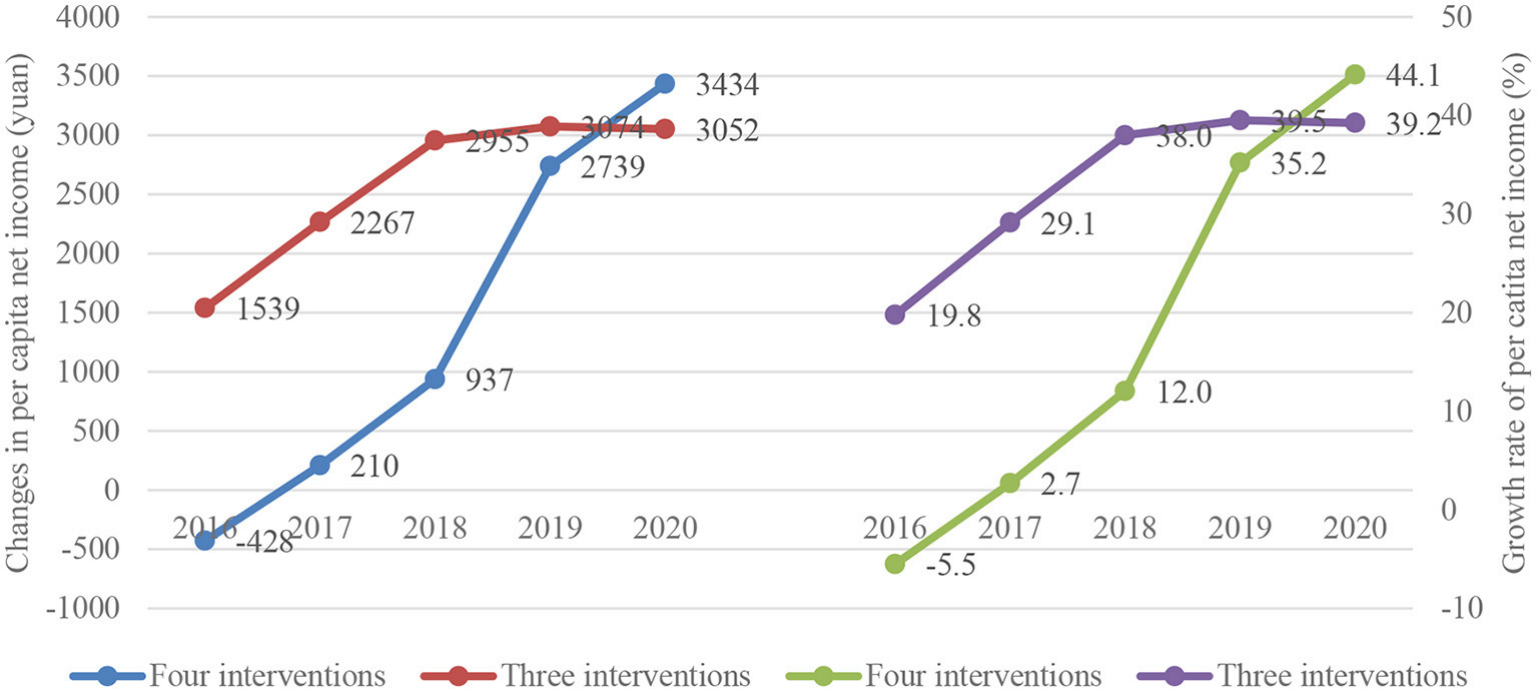
Yearly changes in per capita net income of rural residents (left) and its growth rate (right) in counties with all four measures and countries with three measures.

**Figure 2:**
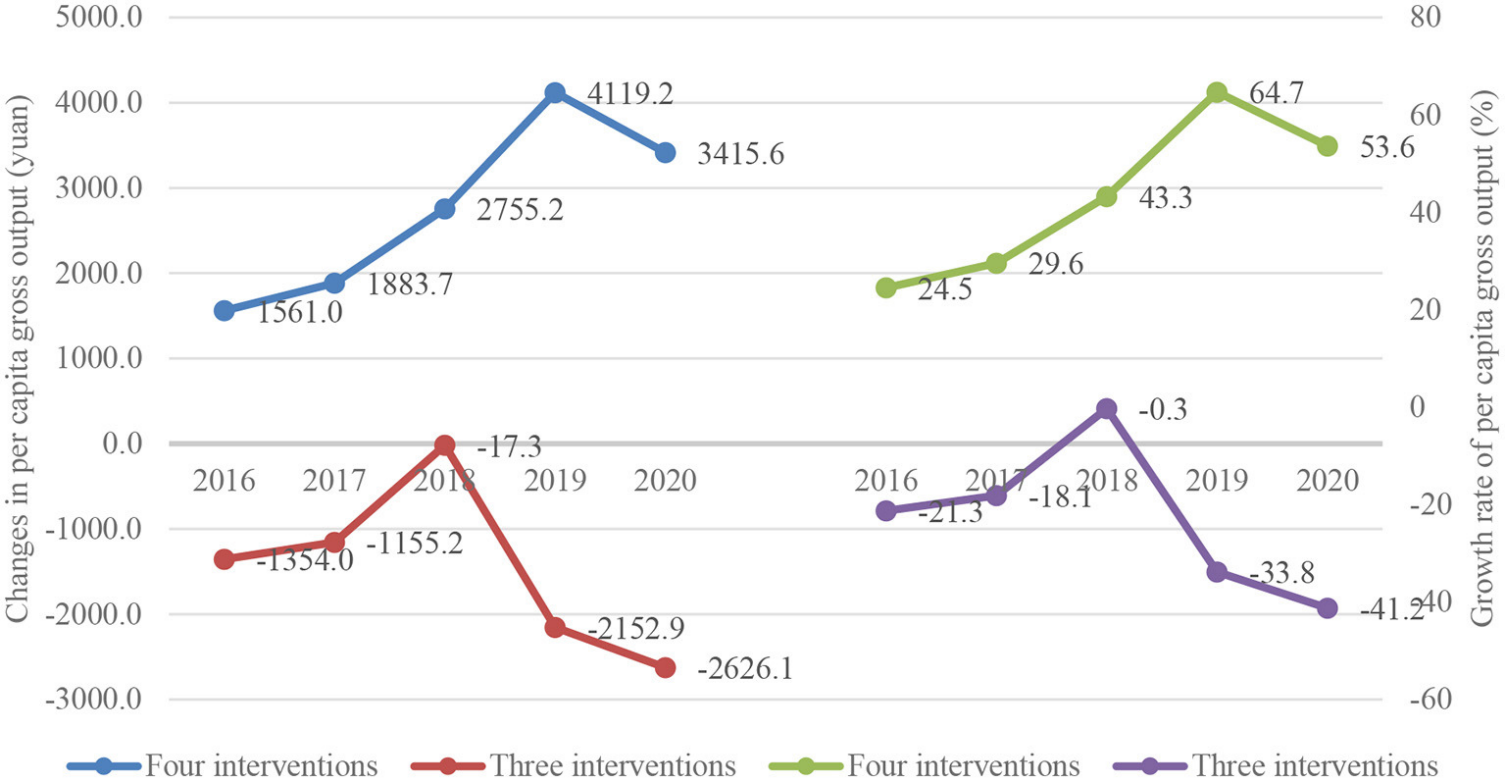
Yearly changes in per capita gross output of animal husbandry (left) and its growth rate (right) in counties with all four measures and with three measures (no lamb vaccination).

Figure 2, tracing the changes in per capita gross output of animal husbandry (left) and its growth rate (right) from 2016 to 2020 in the two groups of counties, depicts a more complex pattern: an upward trend followed by a downward trend (starting from 2018) in both dimensions for both county groups. The downward trends in per capita gross output of animal husbandry for both county groups were presumably caused by the enactment of China's Grassland Ecological Compensation Policy in the 2010s, which aims to protect China's vast grassland by reducing grazing scales of sheep and cattle (18). Yet even with these declines, counties with all four intervention measures outperformed those with only three in per capita gross output of animal husbandry during the entire study period (2016–2020), again suggesting substantial economic gains associated with more intervention measures.

### Econometric analysis: The dynamic difference-in-difference model

While the comparative analyses reported above suggest that echinococcosis interventions were effective at promoting the productivity of animal husbandry and rural income in Qinghai, those analyses did not rule out the influence of potential confounding factors. To more rigorously control for confounding factors in economic impact assessment, we implement a dynamic difference-in-differences (DID) analysis (based on Equation 3) below. Table 4 presents the descriptive statistics of the variables used in the estimation, pooling all county-by-year observations.

**Table 4:** Descriptive statistics of variables used in the analysis, all county-by-year observations (*N* = 223).

**Variables**	**Mean**	**SD**	**Min**	**Max**
**Outcome variables**				
Per capita net income of rural residents (yuan)	9,563.7	2,611.8	4,396.7	18,117.9
Per capita gross output of animal husbandry (yuan)	7,144.1	5,649.8	1,373.6	31,491.9
**Explanatory variables**				
All four measures implemented (1 = yes; 0 = no: “either before 2016 or implemented three measures”)	0.3	0.5	0.0	1.0
**Control variables**				
Proportion of labor at high school or above (%)	11.7	8.8	0.5	50.4
Proportion of illiterate labor (%)	14.9	13.8	0.3	65.7
Proportion of off-farm labor (%)	19.1	12.0	0.2	49.1
Number of villages with roads	105.3	105.8	7.0	380.0
Number of villages with telephone	100.5	108.2	1.0	380.0
Number of villages with running water	98.3	107.1	1.0	380.0

Source: author's calculation.

#### Parallel-trend test

Before presenting the main results of estimating our dynamic DID model (Equation 3), it is helpful to assess the validity of the parallel-trend assumption. Table 5 reports the results of estimating Equation (4) for both economic indicators of interest, i.e., per capita net income of rural residents (columns 1–2) and per capita gross output of animal husbandry (columns 3–4).^7^ Reassuringly, the estimated coefficients of $D_{is}^{0}\times d_{2015}$ are statistically insignificant in all columns, with or without control variables, suggesting no significant treatment-comparison differences in the time trend of either outcome indicator before the intervention. These results lend strong support to the parallel-trend assumption.

**Table 5:** Results of testing the parallel trend assumption.

**Variables**	**(1)**	**(2)**	**(3)**	**(4)**
	**Per capita net income of rural residents**	**Per capita net income of rural residents**	**Per capita gross output of animal husbandry**	**Per capita gross output of animal husbandry**
Treatment ($D_{is}^{0}$) × Year 2015 (*d*_2015_)	−38.16	−134.83	−53.66	186.68
	(1,112.94)	(862.76)	(2,207.53)	(1,967.34)
Year 2015	488.05	467.73	−104.34	−246.22
	(940.60)	(729.39)	(1,865.71)	(1,663.23)
Treatment	166.91	1,274.36	5,008.17^***^	3,055.57
	(786.96)	(828.95)	(1,560.96)	(1,890.24)
Control variables	No	Yes	No	Yes
Constant	7,301.71^***^	5,117.14^***^	2,628.79^*^	8,898.94^***^
	(665.11)	(1,181.68)	(1,319.25)	(2,694.57)
*N*	70	70	70	70
*R* ^2^	0.014	0.463	0.236	0.450

Control variables include the proportions of labor at high school or above, illiterate labor, and off-farm labor, and the numbers of villages with roads, with telephones, and with running water.

Robust standard errors are clustered at the county level and appear in parentheses.

^***^, ^**^, and ^*^ indicate statistical significance at the 1, 5, and 10% levels, respectively.

#### Main results

Table 6 reports the main results of estimating our dynamic DID model (Equation 3) for the two economic indicators of interest, with various specifications. Using rural residents' per capita net income as the outcome variable, column (1) presents the simplest DID model with no control variables. The results indicate that relative to counties that implemented only three intervention measures (without lamb vaccination), those implementing all four measures enjoyed a gain in per capita rural net income by 1,294 yuan on average, an impact statistically significant at the 1% level. As more control variables are added to the model (columns 2–6), the estimated gain in per capita income remains stable (ranging from 1,254 to 1,372 yuan) and highly statistically significant, suggesting that our dynamic DID model is reasonably well-specified.

**Table 6:** Regression results of dynamic difference-in-differences models.

**Outcome variables**	**(1) Per capita rural net income**	**(2) Per capita rural net income**	**(3) Per capita rural net income**	**(4) Per capita rural net income**	**(5) Per capita rural net income**	**(6) Per capita rural net income**	**(7) Per capita gross output of animal husbandry**
Implemented all four intervention measures	1,294.48^***^	1,254.38^***^	1,262.97^***^	1,320.61^***^	1,323.59^***^	1,372.48^***^	938.53^***^
	(219.665)	(236.453)	(245.367)	(271.835)	(268.755)	(239.436)	(190.225)
Proportion of labor at high school or above		66.64^*^	69.08^*^	67.22^*^	87.15^*^	80.57^*^	97.66^**^
		(37.904)	(37.325)	(38.977)	(44.276)	(46.058)	(38.665)
Proportion of illiterate labor			18.13	22.15	32.71	48.38	−17.71
			(45.388)	(46.031)	(47.103)	(44.304)	(22.514)
Proportion of off-farm labor				28.60	28.42	27.62	58.78^***^
				(31.377)	(31.500)	(33.150)	(17.336)
Number of villages with roads					−5.50^*^	−47.13^**^	−11.74
					(2.948)	(21.007)	(8.022)
Number of villages with telephone						16.23	0.84
						(23.477)	(6.972)
Number of villages with running water						26.34^*^	−7.53
						(13.542)	(5.791)
Year fixed effects	Yes	Yes	Yes	Yes	Yes	Yes	Yes
County fixed effects	Yes	Yes	Yes	Yes	Yes	Yes	Yes
*N*	223	223	223	223	223	223	223
*R* ^2^	0.484	0.495	0.496	0.500	0.503	0.519	0.668

Robust standard errors are clustered at the county level and appear in parentheses.

^***^, ^**^, and ^*^ indicate statistical significance at the 1, 5, and 10% levels, respectively.

Turning to the second economic indicator of interest, column (7) (with the largest DID model) suggests an economic gain of 938 yuan in per capita gross output of animal husbandry as a result of implementing all four measures rather than only three. Recall that the trajectories of the two economic indicators of interest are quite different during the project period (2016–2020) (Figures 1, 2). Yet despite this difference, the similarity in the dynamic DID estimates of the economic gains from echinococcosis interventions suggests that our dynamic DID model performs reasonably well in controlling for confounding factors (such as those leading to the different trajectories of the outcome indicators).

#### Heterogeneity

To further analyze whether the economic gains from echinococcosis interventions differed across counties with different infection levels and region types, we re-estimated the dynamic DID model for subsamples of counties defined by different values of the above characteristics. Table 7 reports the results. Columns (1)–(2), dividing the full sample by echinococcosis infection levels, reveal a larger gain in per capita rural net income from echinococcosis interventions (lamb vaccination) for counties with infection level-2 than level-1 counties. The gain was 1,132 yuan in infection level-1 counties and 6,491 yuan in infection level-2 counties. Columns (5)–(6) reveal that echinococcosis interventions (lamb vaccination) raised per capita gross output of animal husbandry by 903 yuan in counties with infection level-1 but had no significant impact in counties with infection level-2. Dividing the full sample into two types of regions (pastoral vs. non-pastoral),^8^ columns (3)–(4) and columns (7)–(8) reveal stronger effects on per capita net income and per capita gross output of animal husbandry among non-pastoral counties relative to pastoral counties.

**Table 7:** Results of heterogeneity analysis of counties with different characteristics.

**Variables**	**Per capita rural net income**				**Per capita gross output of animal husbandry**			
	**Infection level**		**Area type**		**Infection level**		**Area type**	
	**(1) Level-1**	**(2) Level-2**	**(3) Pastoral**	**(4) Non-pastoral**	**(5) Level-1**	**(6) Level-2**	**(7) Pastoral**	**(8) Non-pastoral**
Four measures or not	1,132.99^***^	6,491.60^***^	1,372.09^***^	3,308.60^***^	903.89^***^	−183.00	913.27^***^	1,035.89^**^
	(195.399)	(1,400.096)	(302.785)	(760.610)	(211.288)	(1,152.517)	(278.011)	(390.137)
Proportion of labor at high school or above	66.98	149.87	35.33	170.95^***^	113.08^**^	51.45	115.13^**^	70.78^**^
	(44.911)	(107.173)	(53.248)	(34.662)	(50.969)	(44.357)	(55.377)	(31.613)
Proportion of illiterate labor	19.34	−337.33	47.55	−56.84	−23.27	153.06	−21.03	95.33
	(35.158)	(203.628)	(40.692)	(182.810)	(25.801)	(137.356)	(25.251)	(74.085)
Proportion of off-farm labor	−1.77	−17.39	51.28	−59.49^*^	33.59^**^	100.67^*^	41.06^**^	116.79^***^
	(26.476)	(50.725)	(55.832)	(30.455)	(12.247)	(41.180)	(15.875)	(31.121)
Number of villages with roads	−36.46^*^	−80.67	−36.32^*^	−29.52	−15.88	−30.35	−5.22	−15.79
	(18.218)	(67.750)	(21.058)	(32.372)	(12.117)	(19.132)	(7.799)	(16.293)
Number of villages with telephone	−7.46	262.72	31.84	17.14	−5.96	29.90	6.93	−9.76
	(16.728)	(155.078)	(37.210)	(11.722)	(8.719)	(17.013)	(11.547)	(9.201)
Number of villages with running water	41.51^***^	−187.74	60.10	4.54	−5.02	−12.60	−8.95	6.00
	(14.798)	(121.084)	(45.239)	(22.743)	(5.768)	(12.319)	(15.803)	(8.579)
Year fixed effects	Yes	Yes	Yes	Yes	Yes	Yes	Yes	Yes
County fixed effects	Yes	Yes	Yes	Yes	Yes	Yes	Yes	Yes
Constant	7,857.89^***^	11,608.75^***^	4,119.50^**^	10,646.22^***^	8,451.73^***^	1,053.44	8,391.01^***^	1,347.23
	(718.712)	(2,898.050)	(1,949.359)	(1,543.044)	(733.465)	(2,366.914)	(1,143.135)	(1,018.943)
*N*	181	42	133	90	181	42	133	90
*R* ^2^	0.635	0.697	0.518	0.673	0.734	0.563	0.713	0.671

Robust standard errors are clustered at the county level and appear in parentheses.

^***^, ^**^, and ^*^ indicate statistical significance at the 1, 5, and 10% levels, respectively.

## Discussion and conclusion

Using a panel dataset on 39 counties in China's Qinghai province, this study assessed the economic gains from echinococcosis interventions implemented by the *Action Plan for Prevention and Control of Echinococcosis in Qinghai Province (2016–2020)*. Exploiting temporal and spatial variations in the number of intervention measures implemented, we conducted a series of comparative analyses of economic gains across different counties. To further control for the influence of potential confounding factors, a more rigorous dynamic difference-in-differences analysis was performed to gauge the economic impact of echinococcosis interventions (in particular, lamb vaccination). To the best of our knowledge, this study is the first systematic assessment of the economic impact of echinococcosis interventions in China, if not the first in developing countries.

Although standard echinococcosis intervention measures, including dog management and deworming, lamb vaccination, slaughter management, and training and public education, have been found to be effective and economically viable in curbing the spread of echinococcosis in other countries, they have not been systematically implemented in Qinghai until 2016. Even during the *Action Plan (2016–2020)*, only about half of the counties in Qinghai implemented all four abovementioned measures; the other half implemented only three (without lamb vaccination).

For lamb vaccination, the major challenge is its high cost and the resistance from farmers who fail to see the detrimental effects of echinococcosis on their herd, their livelihood, and even the entire livestock sector and the ecosystem (23, 24). In Qinghai, the cost of lamb vaccination was paid by the central government, but due to the government's limited financial resources, vaccines were distributed only to remote pastoral counties where echinococcosis was most severe (e.g., Yushu and Guoluo Counties). While a number of studies and field trials conducted in other countries have demonstrated the effectiveness of lamb vaccination (24, 25), it remains unclear whether this measure will be effective in China, especially whether it can generate sufficient economic gains to incentivize relevant parties to embrace it. The present study serves to fill these gaps. Our findings, we believe, help inform public policy regarding echinococcosis prevention and control in China.

Our findings provide evidence that echinococcosis interventions implemented by the *Action Plan (2016–2020)* generated substantial economic gains. Our comparative analysis and the dynamic difference-in-differences analysis revealed that all economic indicators examined, including per capita net income of rural residents, per capita gross output of animal husbandry, and their respective growth rates, were significantly higher in the counties with all four intervention measures implemented than those with only three. These findings are consistent with previous analyses conducted in Sweden, which showed that the Swedish entry rules for compulsory pet deworming provided more benefits to society than no rules (26). Our findings suggest that more intervention measures help reduce echinococcosis infection and death rates in livestock, as well as the costs of diagnosis, treatment, and prevention of related diseases. The resulting increases in livestock production capacity will likely generate higher revenues for the region. Revenues may also rise as a result of the higher prices of healthier livestock products sold to the market.

We also found that echinococcosis interventions generated greater economic gains in non-pastoral counties and counties with infection level-2 than in pastoral and infection level-1 counties. One possible reason is that rural residents in remote pastoral areas (where echinococcosis infection is more severe) lack knowledge about the life cycle of echinococcosis, its contamination routes, and its effects on livestock. The lack of knowledge contributed to the high echinococcosis prevalence in such areas and may have reduced the potential effect of echinococcosis interventions (24, 27, 28). Even though one of the measures implemented in Qinghai was the promotion and training of echinococcosis prevention and control practices, with publicity materials written in both Chinese and Tibetan, the scope of promotion and training in pastoral counties was much smaller than in non-pastoral counties due to the former's remoteness and isolation.

Another reason is that training was mostly targeted at men (24). Given women's good habits, such as treating water, washing hands, and adopting other good hygiene practices, that can effectively reduce the risk of echinococcosis infection, women being less informed remains a major obstacle to improving the effectiveness of echinococcosis prevention and control (29). It follows that women should be a primary target in later publicity and training interventions, and relevant policies should overcome their exclusion by stressing the crucial role of the association between their awareness, good habits, and the effectiveness of echinococcosis prevention and control.

The final reason is that it takes farmers in pastoral counties longer to take the livestock to market than in those non-pastoral counties due to inconvenient transportation and the traditional habit of keeping livestock as pets until their death.

Before closing, a note on the limitations of our study is in order. First, because our study is conducted based on county-level data from a single province, its findings may not be generalizable to other regions of China. Second, the county-level analysis lacks the power to infer individual farmers' behavior. Future research examining how farmers respond to echinococcosis interventions is likely to be fruitful. Finally, constrained by our data structure, in which some sampled counties implemented all four intervention measures while others implemented three (without lamb vaccination), we were only able to rigorously quantify the economic impact of lamb vaccination but not that of other measures. Future research estimating the economic impact of other measures will also be valuable.

## Data availability statement

The raw data supporting the conclusions of this article will be made available by the authors, without undue reservation.

## Ethics statement

Ethical review and approval was not required for the animal study because this study uses herd level data, and does not deal with animal ethics.

## Author contributions

JC, KY, QC, QZ, JL, SW, LY, and YL designed the study, analyzed data, and edited the manuscript. JC, QZ, JL, and YL collected the data. JC, KY, LY, and YL drafted and statistical analyses. QC and YL made critical revision. All authors contributed to the article and approved the submitted version.

## Footnotes

^1^Source: https://www.chinacdc.cn/mtbd_8067/201607/t20160715_132225.html (accessed 8 October 2022).

^2^http://www.qhnews.com/newscenter/system/2021/12/26/013499763.shtml (accessed 8 October 2022).

^3^$1 = 6.6 yuan in 2011.

^4^Source from National Bureau of Statistics: https://data.stats.gov.cn/easyquery.htm?cn=C01 (assessed: July 15, 2022).

^5^In China's land use zoning system, agricultural zones refer to areas where agricultural production is dominantly crop production; animal husbandry in such areas mainly takes the form of house feeding, with short-term stubble grazing after crop harvest. By contrast, pastoral areas are areas where animal husbandry, usually based on grazing in grassland and desert areas, is the dominant form of agricultural production. Agro-pastoral areas are areas where crop production and animal husbandry both contribute significantly to rural residents' livelihoods.

^6^Specially, the increases were 1,539, 2,267, 2,955, 3,074, and 3,052 yuan, respectively, from 2016 to 2020 for three-measure counties. The increases were −428, 210, 937, 2,739, and 3,434 yuan, respectively, from 2016 to 2020 for four-measure counties.

^7^Four counties dropped due to missing information before the intervention.

^8^As all counties in agricultural areas implemented three intervention measures, the samples from agricultural and agro-pastoral areas are combined into non-pastoral areas.
